# Cryptococcal Traits Mediating Adherence to Biotic and Abiotic Surfaces

**DOI:** 10.3390/jof4030088

**Published:** 2018-07-29

**Authors:** Emma Camacho, Arturo Casadevall

**Affiliations:** Department of Molecular Microbiology and Immunology, Johns Hopkins Bloomberg School of Public Health, Johns Hopkins University, 615 N Wolfe St Room E5132, Baltimore, MD 21205, USA; ecamach2@jhu.edu

**Keywords:** *Cryptococcus*, adherence, environment, pathogenesis

## Abstract

Several species in the genus *Cryptococcus* are facultative intracellular pathogens capable of causing disease associated with high mortality and morbidity in humans. These fungi interact with other organisms in the soil, and these interactions may contribute to the development of adaptation mechanisms that function in virulence by promoting fungal survival in animal hosts. Fungal adhesion molecules, also known as adhesins, have been classically considered as cell-surface or secreted proteins that play critical roles in microbial pathogenesis or in biofilm formation as structural components. Pathogenic *Cryptococcus* spp. differ from other pathogenic yeasts in having a polysaccharide capsule that covers the cell wall surface and precludes interactions of those structures with host cell receptors. Hence, pathogenic *Cryptococcus* spp. use unconventional tools for surface attachment. In this essay, we review the unique traits and mechanisms favoring adhesion of *Cryptococcus* spp. to biotic and abiotic surfaces. Knowledge of the traits that mediate adherence could be exploited in the development of therapeutic, biomedical, and/or industrial products.

## 1. Introduction

Fungi are often referred as to the “Forgotten Kingdom” because they seem to be an afterthought when people consider pathogenic microbes and focus primarily on viruses, bacteria, and parasites, and yet they are among the most abundant organisms on Earth, representing a critically important group of organisms in the biota of the planet [1]. The most complex and evolutionary members of this kingdom, known as “higher” fungi, are found in the phyla *Ascomycota* and *Basidiomycota*. These phyla include a vast number of species with diverse lifestyles, ranging from wood-decomposing mushrooms, mutualistic mycorrhizae, phytopathogens for agricultural crops, to saprophytic yeasts. Among the soil saprophytes, several *Cryptococcus* species have emerged as major human pathogens [2].

The genus *Cryptococcus* includes about 30 species found ubiquitously in the environment, but two species complexes, *C. neoformans* and *C. gattii* cause most human disease [3]. The disease cryptococcosis follows infection by the inhalation of spores or desiccated yeast cells, and can progress into pneumonia. In immunologically intact hosts, cryptococcal infections are usually contained in the lung, but in the setting of impaired immunity, infection can progress to meningoencephalitis, which is uniformly fatal unless treated. The medical relevance of *C. neoformans* increased dramatically during the late 1970s and early 1980s, when cryptococcosis was found to be a common disease in those with advanced HIV infection [4]. More recently, despite effective introduction of antiretroviral therapy, the incidence of cryptococcal meningitis (CM) remains a challenge even in developed countries. In the USA, its annual incidence is approximately 2–7 cases per 1000 HIV-infected persons with a mortality rate up to 12% [5,6], attributable to limited access to health care [7], and/or late diagnosis of the cryptococcal infection [8]. In 2013 a national trend showing an increased in-hospital mortality among non-HIV CM was reported [9]. In the non-HIV population, some underlying conditions that may act as risk factors for death include advancing age, solid organ transplant recipient, liver disease, renal failure, diabetes, impaired cell-mediated immunity, and others (reviewed by [10]). Significantly higher mortality rates were observed in these HIV-negative patients when compared with their HIV-positive counterpart, e.g., individuals with end-stage liver disease, reported a mortality as high as 80% [11].

Molecular evolutionary studies revealed that *C. neoformans* and *C. gattii* diverged from a common environmental ancestor as recently as 30–40 million year ago [12,13], or as long as 100 million year ago [14]. Given that these species emerged in Africa and South America, respectively, and that the older date correlates with the breakup of the supercontinent Pangea, continental drift has been proposed as the initiating mechanism for speciation [14]. Notwithstanding the spatial and temporal separation, these fungal species show a similar morphology and share virulence factors; but marked differences on their geographical distribution, ecological habitats [15,16,17,18,19] and virulence [20,21,22] have been widely reported. Recent phylogenetic analyses further revealed a high genetic complexity within the *Cryptococcus neoformans/gattii* complex, and proposed a seven-species classification [23]. Extensive biological comparisons between the various species remain to be performed, therefore, for the purpose of this review the basic two-species nomenclature *C. neoformans* and *C. gattii* is used.

Unlike many other human pathogens, *Cryptococcus* spp. require no animal host to survive and complete their life cycle. These fungi interact with free-living amoeba and soil nematodes, which could pose a selective force for fungal factors that are serendipitously advantageous for survival in mammals. Thus, *C. neoformans* and *C. gattii* are facultative intracellular pathogens that have evolved sophisticated strategies for virulence in a broad host range. Their extraordinary ability to survive harsh and fluctuating conditions in vivo could be explained by adaptations to the ecology at the source of infection [24]. These environmentally acquired microbes are exposed to an endless number of stress conditions, which are not necessarily experienced by organisms that are transmitted among vertebrate hosts. Abiotic and biotic pressures, such as temperature, humidity, pH, solar radiation, and competition for nutrients with other soil-dwelling microbes have driven a selective adaptation process favoring *Cryptococcus* spp. saprophytic and intracellular lifestyles. This phenomenon, termed “pre-adaptation” to describe the emergence of a new trait function not immediately beneficial [25], has been also described as “ready-made”, “accidental”, or “stochastic” virulence [26,27], which posits that the pathogenic potential of these fungi is a consequence of adaptation to their normal ecological niche.

## 2. Cryptococcal Adhesion Tools

Cellular attachment is the ability of a single cell to stick to another cell or an extracellular substratum. Cell adhesion also plays important roles in cell communication and regulation of cellular processes, e.g., differentiation, cell cycle, migration, survival [28]. In medical mycology, perhaps the best studied organisms are *Candida* spp. which have many adhesins for interaction with the human host (reviewed in [29,30,31]). For fungal pathogens, adhesins represent a repertoire of cell-wall proteins mediating specific protein-protein, protein-sugar, or other protein-ligand interactions, which are key to the infection process. In saprophytic fungi, cell-wall adhesins promote binding to nutrient-rich surfaces.

**Figure 1:**
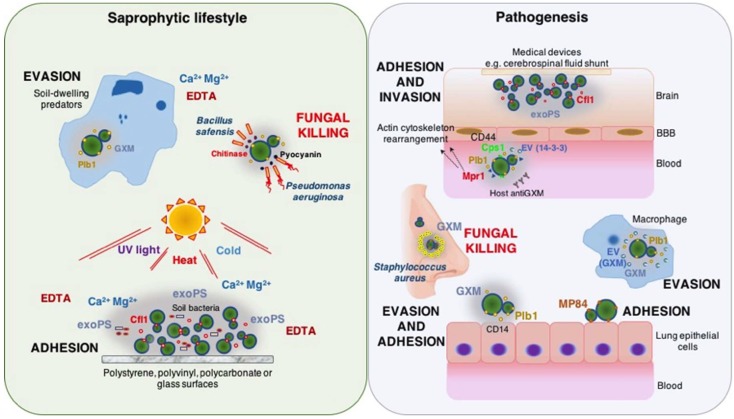
*Cryptococcus* spp. display multiple “cards” of virulence. Unique cryptococcal attributes allow these fungi survival in harsh soil (saprophytic lifestyle) and animal (pathogenesis) environments, as well as binding to abiotic and biotic surfaces promoting nutrient acquisition or tissue colonization and invasion. However, under certain conditions these microbial traits could promote the interaction with other microorganisms that negatively impact fungal viability. In nature, the cryptococcal capsular polysaccharide is a critical component mediating fungal survival amongst soil-dwelling predators. It also allows this microorganism to establish symbiotic communities with other microbes through biofilm formation in response to environmental stressors. In an intracellular setting, cryptococcal yeasts exploit an array of attributes tailored to specific micro-environments. The ultimate aim is overcome the immune defenses and successfully invade host tissues (lung epithelial cells or brain). GXM: glucuronoxylomannan. exoPS, exopolysaccharides (mainly GXM). Plb1: phospholipase 1. MP84: mannoprotein 84. Cfl1: cell flocculin 1. Cps1: hyaluronic acid synthase. Mpr1: metalloprotease. EV: extracellular vesicles. BBB: blood brain barrier.

Unlike the cell wall from most pathogenic yeasts, the cryptococcal cell wall surface is concealed by a polysaccharide capsule that acts as the first component to interact with the outer environment. Hence, there is limited information on cryptococcal adhesins. Nevertheless, many cryptococcal traits could play multiple functions that translate into the capacity for survival and/or animal virulence during their saprophytic or intracellular lifestyles. In a metaphorical way, these fungal pathogens can be seen as holding a unique “hand of cards” when playing the game of virulence [32]. Each card represents an attribute that could promote survival in soil and animal environments, a dual-use card. Depending on the context, some “cards” may render multiple advantages for survival and virulence, or even jeopardize fungal viability (Figure 1). In this regard, the capsule polysaccharide represents a unique and crucial attribute related to adherence and survival of cryptococcal fungi in their natural habitat and in pathogenesis. These capsular attributes are due to extraordinary antiphagocytic properties and ability to promote biofilm formation.

### 2.1. The Capsule Polysaccharide

The cryptococcal capsule is a highly hydrophilic polysaccharide structure (reviewed in [33,34,35]), critical for virulence and it plays a fundamental role in the environment and the animal host. It is mainly composed of glucuronoxylomannan (GXM), which makes up to 90% of the capsule, and two minor components: glucoroxylomannogalactan GXMGal (9–10%), and a small proportion of highly immunogenic mannoproteins (<1%). GXM is a branched polymer of α-(1,3)-mannan with β-(1,2)-xylose and β-(1,2)-glucuronic acid substitutions. These molecular structures occur in both *C. neoformans* and *C. gattii*, but differences in the degree of mannose substitutions and mannose-*O*-acetylation give rise to changes in the polysaccharide three-dimensional structure that translate into differences in its antigenic properties [36,37,38,39]. GXMGal is mostly an export product [40,41,42]. These capsular exopolysaccharides are constitutively secreted to the extracellular medium (~90–95% GXM, 5–10% GXMGal) and can be detected in the serum of patients [43].

The exopolysaccharides, particularly GXM, have been associated with the ability of cryptococcal yeasts to adhere on abiotic surfaces (polystyrene plates and diverse medical devices) [44,45,46,47,48]. *C. neoformans* biofilm formation (reviewed in [49]) is characterized by a compact microarchitecture of growing budding yeast cells internally connected by copious amounts of polysaccharide that uniformly spreads over the plastic support and promotes the formation of microcolonies [44]. In a microtiter plate model, secreted polysaccharide is essential for establishment of an exopolymeric matrix (EPM) and for biofilm formation. However, addition of exogenous polysaccharide to the capsular mutant strain C536 failed to generate a biofilm, suggesting that its cellular production is needed. The importance of GXM for biofilm formation was elucidated in an enzyme-linked immunosorbent assay (ELISA) based on the use of monoclonal antibodies to *C. neoformans* GXM. These antibodies bind to carbohydrate molecules in the capsule and interfere with polysaccharide release from encapsulated yeasts [50]. This assay demonstrated that binding of shed exopolysaccharide to the solid surface was critical to the biofilm formation, as it enhanced attachment of yeast cells via the local release of capsular polysaccharide. Biochemical analyses of the cryptococcal biofilm matrix revealed sugars other than those found in GXM, including glucose, ribose, rhamnose, and fucose, suggesting that the polysaccharide matrix must include other polysaccharides [51]. In addition, scanning electron microcopy and fluorescence imaging of mature *C. neoformans* biofilms revealed a complex structure of flower-like clusters of metabolically active cells interconnected by extracellular polysaccharide material associated with water channels; and, orthogonal images showed a biofilm thickness in the range of 50–76 µm [51,52,53]. Shotgun proteomics for comparative analysis of protein expression from *C. neoformans* biofilms and planktonic cells identified several proteins differentially regulated or unique in the cryptococcal biofilm [54]. Increased expression of enzymes related to proteolysis and protection against oxidative stress was reported, as well as proteins uniquely identified in biofilm such as Cu/Zn superoxide dismutase (CNAG_02852) and acid phosphatase (CNAG_04236), which could have a possible function on biofilm adhesion according to a further interactome analysis.

#### 2.1.1. Interactions of *Cryptococcus* spp. in Soils: A Training Ground for Virulence

Several studies have evaluated *Cryptococcus* spp. interactions with potential single-celled environmental hosts such as *Paramecium* spp. [55], *Dictyostelium discoideum* [56,57], and *Acanthamoeba castellanii* [58,59,60,61,62,63,64]. *A. castellanii*, a fresh water and soil protozoan, is one of the best-studied models for host-parasite interactions due to its extensive association with multiple pathogens (reviewed by [65]). The *Cryptococcus*-amoeba relationship has been described as a symbiotic process, where fungi develop and display escape mechanisms highly useful within macrophages infection; while amoeba enhances its virulence by allopatric gene exchange with their intracellular parasites [66,67]. In fact, there are fascinating similarities in the processes of *C*. *neoformans* infection of *A*. *castellanii* versus macrophages at the cellular level [58,60,68,69,70,71]. The fungus is engulfed and internalized by amoeba and then killed in the phagosome. However, in certain instances *Cryptococcus* can escape its predator via lysis, or by non-lytic exocytosis. Some fungal traits for virulence in mammals play no relevant role for virulence in *A. castellanii* amoeba, such as the alpha mating locus [72] or urease activity [73]. If these anti-mammalian virulence factors are examples of pre-adaptation they must have developed in response to different hostile environmental conditions.

*Cryptococcus* spp. have developed several survival strategies that protect these fungi against predation from soil living microorganisms and even to cope with harsh environmental conditions, such as cold, heat, and ultraviolet light [51,58,74].

Role of the GXM capsular polysaccharide

While studying the interaction of *C. neoformans* with *A. castellanii*, Steenbergen et al. [58] demonstrated that *C. neoformans* was able to grow when co-incubated with amoeba once it has been phagocytosed, while colony forming units of an acapsular strain were significantly reduced as early as 6 h post co-incubation. Incubation of the acapsular strain with exogenous soluble GXM allowed the fungi to form an artificial capsule that protected against predation, although, fungal ability to replicate when incubated with amoeba was not recovered. Consistent with this report, two studies involving *C. neoformans* interaction with soil microbes have shown that capsule enlargement is associated with a significant reduction in phagocytosis [60,75]. Using time-lapse microscopy, Chrisman et al. [60] revealed that *C. neoformans* escapes killing by *A. castellanii* relying on non-lytic exocytosis, a process that is delayed by the presence of polysaccharide capsule. This finding has recently been confirmed and extended to *D. discoideum* [57]. Nonetheless, differences in the capsular GXM structure among cryptococcal yeasts were responsible for different interactions with amoeba; *A. castellanii* phagocytic index for *C. gattii* is lower than for *C. neoformans* [59], which could be attributable to *C. gattii* GXM mannose *O*-acetylation altering the sugar recognition by amoeba phagocytic receptors. While analyzing the interaction between the soil bacterium *Acinetobacter baumannii* and various *C. neoformans* serotype A and serotype D strains, Abdulkareem et al. [75] demonstrated that serotype A strains show higher survival rates than serotype D strains. Increased fungal survival was associated to a two-fold increase in the capsule polysaccharide thickness after exposure to the bacteria. In addition, recent work analyzing *C. neoformans* interaction with *A. castellanii* demonstrated that the fungus shows increased resistance to oxidative and nitrosative stress after exposure to amoeba cells, most likely due to induced alterations on the capsule polysaccharide architecture [62]. Ubiquitous presence of chelating agents such as EDTA in the environment [76], could also impact the cryptococcal-amoeba interaction via removal of calcic and divalent magnesium cations [64,77]. The capsular polysaccharide assembly results from divalent cation-mediated self-aggregation of extracellularly accumulated GXM molecules [77]. These cations increase amoeba predatory capacity by enhancing its attachment on surfaces, thereby increasing contact with *C. neoformans* [64]. Nevertheless, free EDTA degradation by microbial communities could be achieved if complexed with equimolar quantities of calcium and magnesium ions, whose soil availability is associated with content of organic matter particles and pH, among others [78].

Growth inhibition of *Cryptococcus* strains, serotypes A, D, and B, during exposure to *Pseudomonas aeruginosa* was also described [79]. The Gram-negative bacterium-mediated fungicidal effect on *C. neoformans* was dependent on physical contact and production of the quorum-sensing molecule pyocyanin. However, in this study a possible protective effect from the fungal polysaccharide capsule might have been apparent, as a 2-log increased survival of *C. neoformans* was seen when co-culture occurred in DMEM (poor medium) in comparison to YPD (rich medium). The cryptococcal capsule enlargement responds to transcriptional regulation [80] and multiple environmental factors (reviewed by [81,82]). Notably the metabolic stress caused by a low nutrient media was suggested as a key factor for induction of increased capsule in the same strain background used in this work [83,84]. These authors suggested that reduced fungal growth inhibition could have been due to a slow replication of the bacterial strain in the minimal medium. In fact, both theories together could explain the increased resistance of the fungus on the conditions tested.

Role of the cryptococcal biofilm

Cryptococcal biofilm formation provides an adaptation to tolerate and survive hard conditions during the fungal saprophytic lifestyle. Martinez et al. [51] assessed the susceptibility of *C. neoformans* biofilms and planktonic cells to diverse environmental stressors using CFU counting and 2,3-bis(2-methoxy-4-nitro-5-sulfophenyl)-5-[(phenylamino) carbonyl]-2*H*-tetrazolium-hydroxide (XTT) reduction assays to quantify cellular mass and fungal metabolic activity, respectively. Cryptococcal biofilms had increased survival compared to planktonic cells when exposed to a range of temperatures from −80 °C to 47 °C, or UV light doses of 300 µJ × 100/cm^2^. *C. neoformans* biofilms display affinity for a variety of support materials (polystyrene, polyvinyl, polycarbonate, glass); fungal cells formed the strongest biofilms on polyvinyl surfaces, as support roughness promoted adhesion by diminishing shear forces. At a neutral pH milieu, *C. neoformans* strain B3501 also demonstrated increased metabolic activity during the first 24 h and favored a strong biofilm formation.

Several studies have described the interaction between cryptococcal biofilms and other microbes [74,75,85]. Pioneering work by Joubert et al. [74] studied the relationships between a free-living ciliate from the genus *Tetrahymena* and two environmental *Cryptococcus* species (*C. laurentii* and *C. podzolicus*) that are able to form biofilms. These authors reported that biofilm-associated yeast cells are less susceptible to ciliate predation than planktonic cells. Indeed, fluorescent probes showed a symbiotic interaction where protista grazing on the extracellular polymeric matrix (EPM) as nutritional source led to enhanced biofilm metabolism, increased biofilm biomass, and increased fungal cell viability. Biofilm microchannels are critical for directional movement of some soil bacteria, and may support nutrient and gas exchange, while the biofilm structure provides the fungal cells with protection against diverse harsh conditions, from predators, to shear forces, and to desiccation [86,87,88].

The Gram-negative soil predator *A. baumannii* forms mixed biofilms with *C. neoformans* serotypes A and D strains in a microtiter well model that allows chemotactic exchange through the supernatant [75]. *C. neoformans* serotype A strains displayed increased survival, higher capsular polysaccharide production, and a greater number of metabolically active cells within the biofilm than those of serotype D; morphological differences between *C. neoformans* serotypes A and D biofilms were also noticed. Serotype A biofilms were characterized by a uniform distribution of cells throughout the imaged field, while serotype D strains exhibited aggregates of cells scattered in the imaged field. The authors suggested that physical architecture of these biofilms was critical for fungal survival and could reflect an association of a certain serotype to specific environments, e.g., serotype A tight biofilm structure might provide protective advantages when interacting with other microbes or colonizing the human host.

Hypothesizing that microorganisms that shared environmental reservoirs may develop antagonistic mechanisms, Mayer et al. [85] demonstrated that chitinase activity of soil bacterium *Bacillus safensis* strongly inhibited cryptococcal polysaccharide production and de novo formation of biofilms. The inhibition was likely to occur by destabilization of the fungal cell wall architecture; preventing proper retention of polysaccharide capsule components. In consonance with other reports involving cationic molecules from the host, the presence of chitosan (deacetylated chitin) in biofilms dramatically affects the metabolic activity and viability of *C. neoformans* [89]. This effect is plausibly explained by chitosan alteration on the negative charge of the fungal cellular membrane [90], which promotes yeast cells to stay on suspension, thus interfering with surface adherence and cell-cell interactions during biofilm formation.

Interestingly, the development of a mature cryptococcal biofilm involves an intercellular communication process tightly associated with adherence. Studies performed by the Lin group, demonstrated that the cell wall-bound and vesicle-released adhesin Cfl1 (Cell Flocculin 1) promotes flocculation (cell-cell aggregation) and biofilm formation at environmental conditions [91], while in a paracrine manner, it may also act as a regulatory signal within a matrix-connected cryptococcal community [92]. This protein has sequence features described in “typical” adhesins, such as a secretion signal, a Cys-rich region, and a EGF-like chitin-binding domain. *CFL1* is highly induced by the zinc finger transcription factor Znf2, a crucial transcription factor for filamentation [91]. The idea that biofilm growth is promoted by secreted products was also shown in a study describing a quorum sensing (QS) system in *C. neoformans* [93]. Pantothenic acid was demonstrated to be among the bioactive molecules responsible for the QS effect.

#### 2.1.2. Intracellular Pathogenesis

Role of the GXM capsular polysaccharide

The capsular GXM participates in the adherence of *C. neoformans* to the respiratory epithelia (reviewed by [94]). The interaction is characterized by attachment to cellular receptors, followed by entry into host tissue. This process avoids phagocytosis and killing of fungal cells by macrophages, and ultimately provides an alternative intracellular niche for survival [24,95]. At a very early stage of the infection, the capsular polysaccharide inhibits phagocytosis by multiple mechanisms: (i) acting as a physical barrier that blocks recognition of phagocytic receptors with epitopes of the cell wall [96]; (ii) binding to CD14 and toll-like receptor 2 and 4, which causes NF-κB to be translocated to the nucleus inhibiting production of TNFα which, in turn, decreases macrophage activation [97]; and (iii) inducing expression of Fas ligand on macrophage surfaces, which in turn promotes the apoptosis of T cells [98]. However, at 2 h after infection around 40% of the yeast cells are phagocytosed [99]. At this time, the capsular polysaccharide triggers the interaction with different host receptors expressed on multiple phagocytic cells to promote the fungal internalization (reviewed by [100]). Another fascinating mechanism by which the capsular polysaccharide successfully inhibits fungal killing after internalization is by interfering with macrophage metabolism. It involves fungal replication within a damaged phagosome leading to permeabilization of the membrane and exposure of the host cell cytoplasm to toxic products. This microbe-mediated damage led to release and accumulation of polysaccharide-containing vesicles into the macrophage cytoplasm. These vesicular contents may alter osmotic conditions within the cell or interact with host cell products and lead to host cell lysis [99,101]. Capsule enlargement also promotes fungal survival by neutralizing oxidative bursts from phagocytic cells [69].

Recent studies investigating mannosylated polymers revealed that carbohydrate-carbohydrate interactions between mannose residues could confer biophysical advantages for pathogen virulence, pathogen-pathogen interaction, and host-pathogen interaction [102]. Using atomic force spectroscopy, these authors showed that polymers of mannose display strong adhesive forces manifested by self-specific interpenetration (self-latching), which occurred via lateral packing interactions between mannose molecules. Under conditions of nutrient or iron deprivation, as those found by the cryptococcal yeasts in mammalian tissues, capsular polysaccharide enlargement is induced [84,103]. As stated above, mannose molecules from the capsular GXM play a critical role on the immune system recognition. Mannose receptors in the lungs bear terminal mannose residues, whereas in other organs have sialic acid residues [104]. This raises the question of whether mannose distribution on *C. neoformans* and *C. gattii* capsule could contribute to different tropism of these cryptococcal yeasts. Distinct differences in the degree of mannose-*O*-acetylation of the capsule polysaccharide backbone of these cryptococcal species may promote differences on their three-dimensional structure, which in turn could lead to specific mannan-mannan interactions between cells. Moreover, the conformation of monosaccharide assembly into macromolecules allowing polysaccharide fiber extension is still unsolved. To add complexity to the capsular structure, as a natural outcome of the cellular replication, capsular and cell wall remodeling requires constant polymerization and hydrolysis of all its components. This process generates soluble oligosaccharides that could be retained within the capsular network, giving rise to hybrid microenvironments composed of polysaccharide-polysaccharide complexes, which have been previously documented by our research group [105,106].

Conversely, cryptococcal capsular polysaccharide interactions with other microbes found in mammalian hosts can have a negative impact on *C. neoformans* survival. *Staphylococcus aureus*, a common microbe isolated from the nasal cavities of healthy individuals, adheres to the capsular GXM. There was 90% killing of the fungi when both organisms were co-cultured in a liquid medium, while the acapsular *C. neoformans* mutant strain Cap67 survived [107]. In situ TUNEL analysis demonstrated DNA fragmentation of *C. neoformans* cells clumped with live bacteria. Addition of native polysaccharide at high concentrations (10 mg/mL) reduced bacterial adherence to fungal cells. The protective effect was due to increased bacterial affinity to the capsular polysaccharide. *S. aureus* recognition of *C. neoformans* surface, specifically the α-(1,3)-mannosetriose moiety of the mannan backbone of GXM, is mediated by TPI, a cell surface glycolytic enzyme-lectin, also considered a cell envelope protein [108].

Role of the cryptococcal biofilm

Biofilm-related invasive fungal infections are difficult to eradicate because of increased cellular resistance to antifungals and reduced susceptibility to host defenses [109]. Cryptococcal biofilms often form in implanted medical devices such as cerebrospinal fluid shunts [110]. Biofilms are more resistant to amphotericin B and caspofungin than planktonic cells (reviewed by [111,112]). Two azole antifungal compounds (voriconazole and fluconazole) were completely ineffective against cells in biofilms despite efficacy against planktonic cells [112]. In contrast, amphotericin B and caspofungin altered GXM shedding and accumulation, thus disrupting formation of the exopolysaccharide matrix critical for biofilm formation. It is noteworthy that these effects were observed when the drugs were used above physiological concentrations, at doses much higher than achievable inside intravascular catheter lumens or localized administration. Another study from these researchers reported that *C. neoformans* biofilms are also less susceptible than planktonic cells to oxidative stress molecules produced by immune effector cells [113]; however, positively-charged antimicrobial peptides, including PG-1, β-defensin-1, and β-defensin-3, significantly reduced the metabolic activity of biofilms, possibly due to their increased affinity for the negatively-charged capsular polysaccharide [114].

Furthermore, non-lytic exocytosis after antibody-meditated phagocytosis of *C. neoformans* results in cells expelled as a biofilm-like microcolony. This formation is promoted by antibody-mediated agglutination that subsequently continues to replicate as biofilm [115]. Likewise, phenotypic switching (reviewed by [116]), a mechanism characterized by the generation of new colony variants was enhanced in cryptococcal biofilms compared to planktonic cells grown at 37 °C [117]. The smooth phenotype of *C. neoformans* displayed the highest degree of both adhesion and biofilm formation over the mucoid and wrinkled switch variants. Analyses on the polysaccharide capsule of switch variants revealed that besides altered capsule size, morphological switching also lead to biochemical and biophysical properties of the capsular polysaccharide [118,119].

*C. neoformans* biofilm-like structures known as cryptococcomas play a crucial role for the successful colonization of the host central nervous system (CNS) (reviewed by [120]). Following invasion of the brain parenchyma, the biofilm-like adaptation is favored by slowed proliferation due to cell cycle regulation (G2-arrest) [121], reduced protein synthesis and energy acquisition from a fermentation pathway rather than the tricarboxylic acid cycle [54], which allows fungal survival under oxygen-limited conditions.

As noted above, the chelating agent EDTA not only affects the capsular polysaccharide assembly [77], it also inhibits GXM release into the EPM and, therefore, biofilm formation. Nevertheless, synergism of EDTA with antifungal agents was not seen against *C. neoformans* biofilms [122]. Similarly, GXM-binding mAbs prevented biofilm formation by interfering with GXM shedding [44], but it had antagonistic effects with antimicrobial drugs. A possible mechanism is mAb-dependent formation of a protein layer upon binding to extracellular polysaccharide that prevents drug penetration [123].

### 2.2. Mannoprotein 84 (MP84)

*C. neoformans* releases mannoproteins (MP), proteins often containing 80–90% mannose by mass. These glycoproteins stimulate T-cell responses due to their activation of mannose receptors on dendritic cells (reviewed by [124]). Mannoproteins compose less than 1% of the cryptococcal capsule mass [36,125], and are found primarily in the inner region of the capsule [126,127], closely associated to the cell wall via covalent or non-covalent interactions with β-glucans linkages [128] or by forming disulfide bonds with covalently-bound polypeptides to structural glycans [129].

MP84, is a polysaccharide deacetylase that contains a putative glycosylphosphatidylinositol (GPI)-anchor motif in the C-terminal portion and has a potential site for heavy *N*-glycosylation characterized by the presence of serine/threonine-rich region [130]. MP84 mediates adhesion of *C. neoformans* yeasts to lung epithelial cells in capsule-independent manner [131]. When recombinant MP84 was incubated with capsulated (NE-241) and capsular-polysaccharide defective (CAP67) *C. neoformans* strains, MP84 did not inhibit adherence of the encapsulated strain but blocked the interaction of the poorly encapsulated cryptococcal yeasts. This work suggested a role for MP84 early in infection favoring adhesion of acapsular cryptococcal yeasts to epithelial cells. The role of MP84 as a cryptococcal adhesin during the saprophytic life style has not been explored.

### 2.3. Phospholipase B (Plb1)

Phospholipase B is a GPI-linked cell-wall associated protein contributing to virulence of both *C. neoformans* and *C. gattii* in mammalian hosts. Plb1 is covalently linked via β-(1,6)-glucans to β-(1,3)-glucans and is critical for maintaining the cell wall integrity and fungal survival under amoebic predation or the presence of cell wall stressors such as SDS and Congo red [58,132]. Plb1 has been localized to the cryptococcal plasma membrane associated with lipid rafts, on the cell wall, and also secreted from the cell surface [132,133,134].

During cryptococcal pathogenesis, Plb1 is implicated in multiple stages including: initiation and persistence of the pulmonary infection; fungal escape from macrophages; and, dissemination to the CNS. This protein has three enzymatic activities: phospholipase B (PLB), lysophospholipase (LPL) and, lysophospholipase transacylase (LPTA). Using selective chemical inhibitors and polyclonal antibodies against Plb1, Ganendren et al. [135] demonstrated that PLB activity facilitates adhesion of cryptococci to lung epithelial cells. Addition of palmitic acid led to a dose-dependent increased adhesion of a *plb1* mutant strain to a lung epithelial cell line. Therefore, a possible mechanism for Plb1 role in cryptococcal adherence might involve the enzyme activity on dipalmitoyl phosphatidylcholine (DPPC), the main component of the outer leaflet of plasma membranes and also lung surfactant, generating glycerophosphocholine and free palmitic acid that enhance binding of fungal cells via fatty acid release from host substrates.

Other studies have reported that Plb1 promotes cryptococcal proliferation and survival within macrophages favoring fungal evasion of the immune system. Plb1 is maximally active under acidic conditions, including those of the phagosome [136], and its activity can have a variety of effects: (i) Plb1 may have a role during budding via modification of membrane phospholipids driving fungal intracellular proliferation within the phagocytic cells; (ii) Plb1contributes to cell size changes both in vitro and in vivo [137]; and (iii) Plb1 degrades macrophage membranes to promote phagolysosomal membrane permeabilization, macrophage apoptosis, and fungal escape via lytic exocytosis [138].

Moreover, *C. neoformans* Plb1 activates multiple host signaling events leading to binding and invasion of human brain microvascular endothelial cells (HBMEC) monolayers, fungal transmigration across the blood-brain barrier, and colonization of the brain [139,140]. The process involves rearrangement of the host actin cytoskeleton by activation of three members of Rho GTPases (RhoA, Rac1, and Cdc42), followed by downstream phosphorylation of focal adhesion kinase (FAK), ezrin, and protein kinase C α (PKCα). A *plb1* mutant strain showed significantly reduced cryptococcal binding, invasion, and monolayer transmigration. These phenotypes are mimicked by pharmacological inhibition of these host signaling proteins.

### 2.4. Hyaluronic Acid Synthase

The cryptococcal *CPS1* gene encodes a hyaluronic acid synthase (glycosyltransferase) that shares homology to the type 3 polysaccharide synthase gene, *CAP3B*, of *Streptococcus pneumoniae*. The bacterial gene is essential for synthesis of bacterial capsular lipopolysaccharide [141]. A *CPS1* deletion highlighted ultrastructural changes between the cell wall and capsule, reduced content of hyaluronic acid, and led to fungal inability to associate with HBMEC in vitro.

Fluorophore-assisted carbohydrate electrophoresis (FACE) analysis has detected nanogram quantities of glycosaminoglycan-derived products, revealing hyaluronic acid molecules present in the cryptococcal capsular polysaccharide [142]. These authors reported that hyaluronic acid forms fibrous structures that extend from the cell wall to the exterior part of the yeast cells. Treatment of several *C. neoformans* strains with hyaluronidase, or using a drug that blocks hyaluronic acid production, demonstrated decreased ability to bind to HBMEC in a dose-dependent manner. Other studies demonstrated that *C. neoformans* invasion of brain endothelial cells required the specific interaction between hyaluronic acid on the fungal surface and CD44-containing lipid rafts expressed by HBMEC. This interaction promoted the reorganization of the host cytoskeleton, formation of F-actin-mediated membrane ruffling and lamellipodia-like structures, and migration across the blood-brain barrier (BBB) [143,144,145].

### 2.5. Metalloprotease (Mpr1)

Another cryptococcal component relevant for CNS invasion is the extracellular fungalysin metalloprotease, Mpr1, a member of the M36 class of fungal-specific metalloproteases encoded by the *MPR1* gene [146]. Mpr1 mediates selective attachment and internalization of *C. neoformans* to the BBB both in vitro and in vitro, most likely by enhancing its permeability through disruption of surface proteins of the HBMEC. Its expression into the non-pathogenic yeast *S. cerevisiae* permitted brain infection [147]. Recent analyses including transcytosis in vitro assays, proteomics and microscopy studies, showed that *C. neoformans* transcellular movement and exocytosis mediated by the Mpr1 activity required host Annexin 2 (AnxA2) for inducing the re-organization of the cytoskeleton. AnxA2 is a signaling protein related to multiple intracellular process such as membrane trafficking, endocytosis, and exocytosis. Interestingly, a lack of AnxA2 in vitro function increased fungal adhesion and internalization to the brain endothelial cells, but significantly reduced its partner protein S100A10, which led to fungal entrapment within cellular cytoplasm [148,149]. In fact, AnxA2 was also associated with *C. neoformans* non-lytic exocytosis from macrophages [150].

### 2.6. 14-3-3 Adhesin

In fungi, the 14-3-3 proteins are a family of highly conserved polypeptides that participate in a variety of cellular processes such as signal transduction, cytokinesis, cell cycle regulation, transcription, protein trafficking/secretion, and virulence. These assemble as stable dimers that lack catalytic activity and are able to interact with partner proteins through binding of phosphorylated serine and threonine residues, thus 14-3-3 proteins are considered as small adaptors (reviewed by [151]). In *C. albicans*, 14-3-3 protein plays a fundamental role in both vegetative growth and filamentation, suggesting its association with regulatory pathways required for colonization and invasion [152]. In the fungal maize pathogen *Ustilago maydis*, a basidiomycete like the cryptococcal yeasts, these proteins participate in the cell cycle regulation, cytokinesis, chromosome condensation, and vacuole formation [153].

In *C. neoformans* the 14-3-3 protein (CNAG_05235.1) is found in secreted vesicles, also referred as “virulence bags” due to their ability to transport virulence factors across the fungal cell wall. Vesicle-transported virulence factors include the capsular polysaccharide GXM, laccase and urease, each with potential to modulate the host-pathogen interaction [154]. Li et al. [155] engineered a *C. neoformans* copper-repressible 14-3-3 strain and showed that this protein was involved in *C. neoformans* adherence to HBMEC. That study also showed that 14-3-3 played several roles relevant to *C. neoformans* growth, morphology, cell division, and possibly in secretory/trafficking pathways. Reduction of the 14-3-3 protein levels led to decreased vesicle secretion, which correlated with reduction of capsule size. Other studies revealed that *C. neoformans* vesicles are distributed inside and around CNS lesions, particularly facilitating fungal adhesion to HBMEC and transcytosis [156], further establishing a critical role of cryptococcal vesicles during brain infection.

## 3. Conclusions

A Pubmed search revealed that over 50% of the studies concerning fungal adhesion have been performed on the ascomycetous yeasts *Candida* and *Saccharomyces*, whereas research related to basidiomycetes barely comprises 2% of published articles [157]. Numerous cell-wall adhesins have been shown to be vital for the infection process of human pathogenic fungi, but for cryptococcal yeasts such adhesins are buried under the polysaccharide capsule and these fungi use different mechanisms to accomplish the same goal. Unique microbial attributes involved in adhesion of cryptococcal yeasts during the environmental lifestyle and pathogenesis are summarized in Table 1. As is evident throughout this essay, the polysaccharide capsule is a major component mediating the interaction of cryptococcal yeasts with their environment, including interactions that result in attachment. While reviewing the compiled literature, some intriguing questions came to mind about this particular trait: Could cryptococcal yeasts have “directional movement” along mannosylated surfaces promoted by strong adhesive forces between mannose molecules (self-latching)? Does the polysaccharide capsule favor *Cryptococcus* dissemination in a fluid environment (bodies of water or even bloodstream) while decreasing adhesiveness? How do biofilms, a highly hydrophilic structure, cope with desiccation? Although we do not have answers to these questions, they pose potentially interesting areas for future investigation.

Common, but not universal, features identified in “typical” adhesins include: a secretion signal, high Ser/Thr content, Cys-rich domains, tandem repeats, *N*- and *O*-glycosylations, GPI anchors, dibasic sequence motifs (KK, RR, KR, RK), and potential amyloid-forming β-aggregation segments (reviewed by [157]). These molecules mediate fungal cells interaction with proteins and glycans present on homologous cells, host cells, and abiotic substrates. Ligand binding, hydrophobic effect, and amyloid-like protein-protein interactions are among known binding mechanisms. Identification of fungal adhesins through proteomic studies of biofilms have turned into an excellent tool [158]. Future approaches to identify putative fungal adhesins might involve genomic-level screenings coupled to cross-validation using cell adhesion arrays based on well-established models [157].

Since severe fungal infections have become more common with the widespread use of indwelling medical devices, broad-spectrum antibiotics, and increased immunocompromised patient populations, future research on fungal adhesins during biofilm development would be highly significant to the medical field. Strikingly, a recent study performed by our group demonstrate that *C. neoformans* and *C. gattii* species complex strains conserved the same intracellular pathogenic strategies despite having separated around 100 million year ago, suggesting that these mechanisms are possible maintained by similar selective forces through ages [159]. Hence, particular focus on expanding our understanding of the “atypical” mechanisms related to cryptococcal adherence could be potentially valuable in therapy.

## Figures and Tables

**Table 1:** Cryptoccocal adhesion tools to biotic and abiotic surfaces.

Cryptococcal Attributes	Adherence-Related Roles	
	Saprophytic Lifestyle	Pathogenesis
**GXM capsular polysaccharide**		Binding to respiratory epithelia through interaction with CD14 and TLR2, and TLR 4 receptors [94,97]. Biofilm formation favoring: adherence to intracranial medical devices [110], increased resistance to antifungal drugs [111,112], less susceptibility to host oxidative stress molecules [113], and colonization of the host CNS [120].
**Exo-polysaccharides**	Biofilm formation promoting attachment to abiotic surfaces [51].	
**Mannoprotein 84 (MP84)**		Adhesion to lung epithelial cells in a capsule-independent manner [131].
**Phospholipase B (Plb1)**		Adhesion to lung epithelial cells via fatty acid released from host substrates [135]. Binding to HBMEC monolayers mediated by alteration of the host actin cytoskeleton, which leads to transmigration across BBB, and colonization of the brain [139,140].
**Hyaluronic acid synthase (CPS1)**		Interaction with CD44-containing lipid rafts expressed by HBMEC that promotes rearrangement of the host cytoskeleton, migration across BBB, and invasion of brain endothelial cells [143,144,145].
**Metalloprotease (Mpr1)**		Selective attachment and internalization to BBB via disruption of surface proteins of the HBMEC [147].
**14-3-3 adhesin**		Vesicle-secreted protein involved in adherence to HBMEC monolayer possible promoted by other vesicle transported fungal components such as urease [155,156].

CNS: central nervous system HBMEC: Human brain microvascular endothelial cells; BBB: blood-brain barrier.
